# The appearance of cartilage used in deep inferior epigastric perforator breast reconstruction surgery as a calcified mass on CT scan

**DOI:** 10.1259/bjrcr.20150143

**Published:** 2015-06-30

**Authors:** S Deen, R Bedair, C J Daniels, C Swainson

**Affiliations:** ^1^Cambridge University Hospitals, NHS Foundation Trust, Cambridge, UK; ^2^Cancer Research UK, Cambridge Institute, University of Cambridge, Cambridge, UK; ^3^Department of Radiology, University of Cambridge, Cambridge, UK; ^4^Department of Radiology, Pennine Acute Hospitals NHS Trust, Manchester, UK

## Abstract

This paper describes an unusual radiological appearance of implanted cartilage on CT scan in a patient who had recently undergone deep inferior epigastric perforator (DIEP) breast reconstruction surgery following a mastectomy for ductal carcinoma *in situ.* The purpose of this paper is to alert medical practitioners involved with DIEP breast reconstruction surgery, as well as general radiologists, to the possibility of surgically implanted costal cartilage undergoing calcification and then appearing on imaging studies as a malignant process. Information on the patient was gathered from clinical records, imaging reports and pathological samples. A literature search was performed to identify similar cases and the results showed that this occurrence has never before been described and therefore represents an advancement of knowledge about the imaging characteristics of reconstructed breast tissue.

## Clinical background

A 52-year-old female presented to her general practitioner (GP) after she noticed a lump in her right breast that was increasing in size and occasionally tender. 2 years ago, she had a mammogram and was recalled, but further workup revealed normal breast tissue. Otherwise, the patient remained fit and well, with her only medication being amitriptyline for neuropathic pain. She was immediately referred by her GP to the symptomatic breast lump clinic where examination found a single palpable area of glandular tissue in the upper outer quadrant of the right breast and no lymphadenopathy.

The patient went on to have a mammogram that uncovered a 6.6-mm lesion in the right breast that was reported as benign/indeterminate with a mammography score of M2/3. On ultrasound, the lesion was visible at the 10 o’clock position as a 6.6 × 4.4 × 5.6-mm hypoechoic area classified as U3. Guided fine needle aspiration cytology (FNAC) was performed and confirmed a C5 malignancy. The patient then had an ultrasound-guided core biopsy of the area that revealed intermediate-grade ductal carcinoma *in situ* (DCIS). Following the C5 cytological grading, an ultrasound scan of the right axilla with FNAC of the axillary lymph nodes was carried out. The nodal samples showed malignant cells, and the patient was officially diagnosed as having right breast intermediate grade DCIS with C5 cytology.

An MRI scan was requested to confirm a single focus of disease in the right breast, and 2 weeks later the patient underwent right-sided wire-guided wide local excision and axillary node clearance. Histological examination at this time verified a 7-mm grade 3 invasive ductal carcinoma ER+ (oestrogen receptor positive) and HER2+ (human epidermal growth factor receptor 2 positive), with radiologically occult DCIS at the margins. Nottingham prognostic index was calculated as 5.14 and the cancer was listed as T1cN1Mx according to the pTNM classification.

The clinical and radiological findings in this patient led to her having a completion mastectomy of the right breast, performed at the time without reconstruction. The histological report of the breast tissue from mastectomy described extensive residual high-grade DCIS around the previous wide local excision cavity with the largest focus measuring 14.5 mm. There was also associated cancerization of lobules but no evidence of invasive carcinoma. The only complication of the surgery was a moderate amount of right-sided lymphoedema. Subsequent to mastectomy, the patient was started on a course of adjuvant chemotherapy with docetaxel. She was later treated with trastuzumab and letrozole.

Eventually, after over a year, the patient had a delayed deep inferior epigastric perforator (DIEP) flap reconstruction surgery performed. The results of this reconstruction were initially good. The surgical wounds proceeded to heal well and uneventful left breast surveillance mammograms were performed as a part of follow-up.

## Presentation

**Figure 1.**
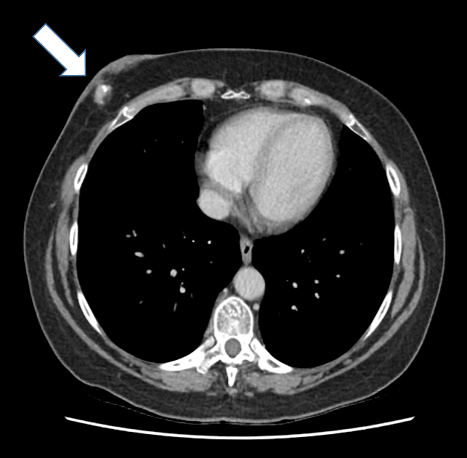
Axial CT slice of the patient’s chest showing the suspicious mass embedded in the right chest wall, at the position of the deep inferior epigastric perforator reconstruction. An area of calcification is visible within the lesion.

The first worrying features began to surface when the patient visited her GP with a 2-month history of cough. A respiratory tract infection was suspected and the appropriate antibiotic therapy initiated. In spite of an adequate treatment course, however, the cough failed to subside. Given her background of cancer, the decision was made to perform an early CT scan of the chest, abdomen and pelvis to look for metastases that could explain the non-resolving cough. No clear spread could be found but the CT scan did uncover one notable abnormality, a 1.7-cm solid calcified lump in the right breast. The relevant CT slice is depicted in Figure 1. No axillary recurrence or mediastinal lymphadenopathy was detected in association with this newly discovered mass. The radiological nature of the lump raised immediate suspicion of a new breast malignancy. It was decided that the patient should be discussed at the next multidisciplinary team (MDT) meeting with a view towards establishing a definitive diagnosis and offering rapid treatment should it be necessary.

## Investigations and differential diagnosis

After input from the MDT, the nature of the structure being visualized still remained unclear. Malignancy had to be considered given the appearance of a calcified mass despite the known rare recurrence of cancer following mastectomy.^1^

**Figure 2.**
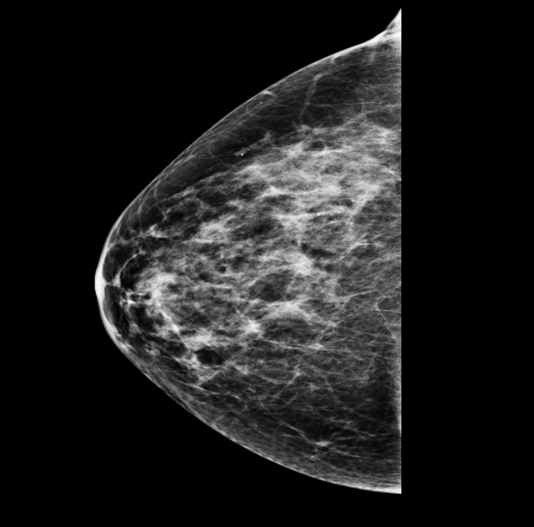
The right breast mammogram taken prior to mastectomy showing the malignant ductal carcinoma *in situ* lesion.

Attempts were made to correlate the mass on different imaging modalities; however, no dedicated post-mastectomy right breast scans were available. The most recent right-sided mammogram had been completed just prior to mastectomy and is illustrated in Figure 2.

**Figure 3.**
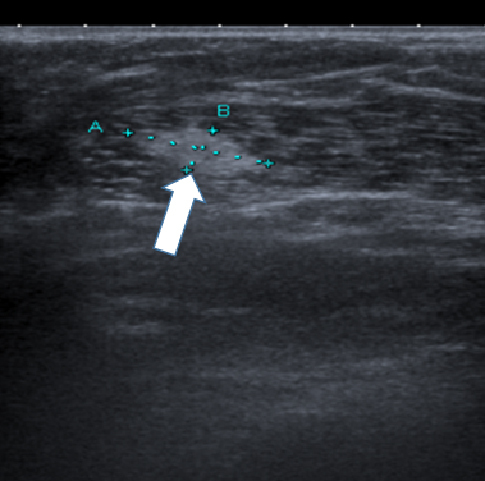
An ultrasound scan of the patient’s chest wall taken to investigate the presence of a palpable lump, later diagnosed as a lipoma. The absence at this time of the calcified lesion later visualized on CT scan is noteworthy.

The only relevant scan available in hospital records for comparison was an ultrasound of the chest wall carried out 2 months after reconstruction to assess the emergence of a soft and mobile subcutaneous mass that was eventually found to be a lipoma. This ultrasound scan had been taken only 4 months before the CT scan and careful review showed a complete absence of the calcified mass now apparent in the breast. A freeze frame of the ultrasound appearance of the right chest wall is shown in Figure 3 where the white arrow points to the lipoma. The approximate time frame revealed by the ultrasound scan for the emergence of the calcified mass raised further suspicion of a rapidly growing tumour.

Another ultrasound scan was performed within a short period of time to further assess the nature of the new right breast lump. It was discovered from this ultrasound scan that the lump contained a significant proportion of cartilage. A review of the case with this new knowledge raised the question of whether the mass might actually represent a transformation of a small piece of costal cartilage implanted into the DIEP flap by the breast surgeon. The CT scan was reviewed to try to identify the position of the cartilage but it could not be found, and so the MDT determined that the most likely way to account for the disappearance of the cartilage and the emergence of a new calcified mass was that the cartilage must have undergone calcification over time and taken on the appearance of a radio-opaque lump.

## Outcome

It was collectively decided at this point that further testing should be avoided. Additional imaging was deemed unnecessary and management of the patient returned to standard of care surveillance following DIEP surgery.

## Discussion

**Figure 4.**
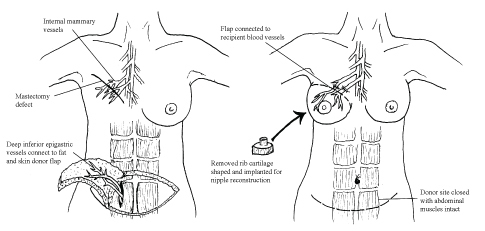
Illustration of the oncoplastic technique in deep inferior epigastric perforator breast reconstruction surgery performed after mastectomy.

DIEP flap breast reconstruction surgery is now a popular option following mastectomy. Advantages include a strong blood supply to the newly implanted tissue, faster recovery time compared with the transverse rectus abdominis myocutaneous flap procedure,^2^ the sparing of donor site muscle,^3^ a reduction in abdominal fat and smaller risk of lymphoedema than with larger operations that typically also involve surgery to the axillary region. ^4,5^ DIEP flap reconstruction is performed with free tissue transfer from the abdominal wall using a microvascular technique. The perforator vessels from the abdominal flap are anastomosed onto the internal mammary vessels that are approached by removing a small portion of rib.^6^ Figure 4 illustrates how the DIEP procedure is performed.

An option for further reconstruction after DIEP is the implantation of the costal cartilage removed while accessing the internal mammary vessels into the DIEP flap. This can later be moulded into the shape of a nipple and has been shown to result in a satisfactory reconstruction for many patients.^7,8^

The appearance of this costal cartilage implanted in the DIEP flap can change dramatically over time. Current literature, however, lacks any description of the changing radiological features of implanted costal cartilage in a DIEP flap when imaged using CT. There are two reasons for this. First, CT scan is not routinely used for post-operative imaging of the breast, and second, when CT scans are taken, they are not done within the time frame required to detect the changes that develop in the implanted cartilage.

After DIEP surgery, some evidence exists for post-operative imaging with CT or MR angiography to assess vascular anatomy and perfusion^9^ but this is as yet far from established practice and would typically take place within a week of surgery. By comparison, the most prominent change to implanted cartilage following DIEP surgery that we have noted in our case is calcification that happens only after several months have passed.

The possibility of cancer returning post-DIEP does exist^10^ and when longer term follow-up imaging is done, the indication is most often to look for recurrence. MRI is the imaging modality of choice in these cases. Calcium deposits do not generate as much contrast on MRIs as they do on CT scans and calcifications, such as the one in this patient, can easily be missed with MRI done to look for recurrent lesions.

The infrequent use of CT scan post-surgery means it can be difficult for clinicians to decipher the unusual and unexpected appearances of implanted costal cartilage when CT scans include portions of reconstructed breast tissue following DIEP surgery. As a consequence of the size and position of implanted cartilage and the typical history of cancer in DIEP reconstruction patients, there is a real risk of misdiagnosis of these masses by radiologists. This case illustrates how without sufficient dissemination of information on uncommon imaging appearances such misdiagnoses can occur and emphasizes the importance of improving awareness about unconventional radiological presentations originating from rarer surgical practices.

## Learning points

1. Radiologists should be alerted to the possibility that suspicious lesions discovered on CT scan after DIEP or other similar breast reconstruction surgeries may simply represent implanted cartilage and therefore not warrant further investigation.
2. Whenever possible, surgeons should attempt to avoid implantations into areas prone to malignant transformation. This will decrease the chances of radiological misdiagnoses and unnecessary interventions.
3. When cartilage or other tissue is implanted into breast, it might be advisable to perform post-surgical imaging to confirm the position and acquire a reference location of the implant for comparison with future investigations.
4. It may be beneficial to radiologists and other clinicians for surgeons to record a precise description of the location of implanted materials in the patient’s records, especially when performing DIEP reconstruction.
